# *N*‐back training and transfer effects revealed by behavioral responses and EEG

**DOI:** 10.1002/brb3.1136

**Published:** 2018-10-23

**Authors:** Valentina Pergher, Benjamin Wittevrongel, Jos Tournoy, Birgitte Schoenmakers, Marc M. Van Hulle

**Affiliations:** ^1^ Laboratory for Neuro‐ & Psychophysiology, Department of Neurosciences KU Leuven – University of Leuven Leuven Belgium; ^2^ Department of Chronic Diseases, Metabolism and Ageing University Hospital Leuven, KU Leuven Leuven Belgium; ^3^ Academic Centre of General Practice, KU Leuven Leuven Belgium

**Keywords:** age‐related differences, *N*‐back task, P300 ERP, transfer effects, working memory training

## Abstract

**Introduction:**

Cognitive function performance decreases in older individuals compared to young adults. To curb this decline, cognitive training is applied, but it is not clear whether it improves only the trained task or also other cognitive functions. To investigate this, we considered an *N*‐back working memory (WM) training task and verified whether it improves both trained WM and untrained cognitive functions.

**Methods:**

As EEG studies have noted task difficulty and age‐related changes in time‐locked EEG responses, called event‐related potentials (ERPs), we focused on the relation between the P300 ERP component, task difficulty level, and behavior response accuracy and reaction time (RT) in young and older healthy adults. We used two groups of young and older healthy participants to assess the effect of *N*‐back training: cognitive training group (CTG) and passive control group (PCG). Before and after training, cognitive tests were administered to both groups to evaluate transfer effects.

**Results:**

Despite the observed age‐related differences in the P300 ERP component and in terms of RT and accuracy, our findings demonstrate a stronger improvement in the trained task for older CTGs compared to younger CTGs, larger near‐ and far‐transfer effect to WM and fluid intelligence for both younger and older CTGs, and a far‐transfer effect to attention but only for older adults. Significant differences in response accuracy were shown between young and older subjects in spatial memory and attention tests.

**Conclusion:**

The application of a WM training is a promising tool for both healthy adults, and in particular for older subjects, as it showed physiological and behavioral differences in cognitive plasticity across life span and evidence of benefits in the trained task and near‐/far‐transfer effects to other cognitive functions.

## 1 INTRODUCTION

Cognitive decline has been sufficiently evidenced in healthy older adults across different cognitive domains (attention, working memory [WM], spatial memory, reasoning) (for review, see Au et al., 2015; Karbach & Verhaeghen, 2014; Soveri, Antfolk, Karlsson, Salo, & Laine, 2017) and, in view of the rapidly increasing elderly population, a growing concern of healthcare organizations in the near future. Given that decline in WM, one of the main cognitive functions, is paralleled by neurochemical, structural, and functional changes in the aging brain (Bopp & Verhaeghen, 2005), the study of cognitive decline during one's life span, and, more importantly, what can be done to slow it down, has gained interest from the research community. Motivated by the alleged ability to rekindle plasticity processes in the brain, cognitive training has been promoted to be effective in improving cognitive function performance after extensive training (Blacker, Negoita, Ewen, & Courtney, 2017; Kundu, Sutterer, Emrich, & Postle, 2013; Yang, Krampe, & Baltes, 2006).

Cognitive training was developed to improve different cognitive functions such as attention, perception, and memory in young and older adults (Heinzel, Rimpel, Stelzel, & Rapp, 2017; Loosli et al., 2016; Mahncke, Bronstone, & Merzenich, 2006; Salminen, Kühn, Frensch, & Schubert, 2016), and to tap into the aging brain's plasticity to improve cognitive functions such as intelligence, episodic memory, WM, and executive functions (Dahlin, Nyberg, Bäckman, & Neely, 2008; Lawlor‐Savage & Goghari, 2016; Yang et al., 2006). Moreover, WM training has been shown to yield beneficial effects in older adults reducing age‐related WM decline (Borella, Carretti, Riboldi, & De Beni, 2012; Borella, Carretti, Zanoni, Zavagnin, & De Beni, 2013; Brehmer, Westerberg, & Bäckman, 2012; Buschkuehl, Jaeggi, & Jonides, 2012; Li et al., 2008; Schmiedek, Lövdén, & Lindenberger, 2010), albeit that only a few cognitive training studies, in terms of *N*‐back training, have shown positive effects across age (Heinzel et al., 2017; Lawlor‐Savage & Goghari, 2016; Loosli et al., 2016). Following a series of studies, Dahlin, Neely, Larsson, Bäckman, and Nyberg (2008), Dahlin, Nyberg, et al. (2008) and Li et al. (2008) reported that training on an *N*‐back task improves WM of both young and older healthy subjects. Although some studies showed more improvements in young subjects compared to older ones, as in the studies of Dahlin, Neely, et al. (2008) and Bürki, Ludwig, Chicherio, and De Ribaupierre (2014), other studies showed more improvements in older healthy subjects than in younger ones (Bherer et al., 2005). Several theories have been formulated to explain this discrepancy: (a) overlap in certain elements of a skill between trained and transfer task (Klingberg, 2010), (b) specific cognitive processes that are required in both training and transfer tasks (Dahlin, Neely, et al., 2008; Dahlin, Nyberg, et al., 2008), (c) the achieved final level of performance (based on frequency, duration, task difficulty level, etc.) of training (Morrison & Chein, 2011). Despite this discrepancy, all studies demonstrate WM plasticity in young (Buschkuehl et al., 2012; Jaeggi et al., 2010; Jaeggi, Buschkuehl, Jonides, & Perrig, 2008) and older adults (Borella et al., 2014; Borella et al., 2010; Heinzel et al., 2017; Loosli et al., 2016), and the possibility to obtain near‐ and/or far‐transfer effects. Although the degree of plasticity varies across studies, the potential of the brain to reorganize itself in response to demands is observed across age (Bialystok, & Craik, 2006; Craik & Salthouse, 2002; Heinzel et al., 2014; Lawlor‐Savage & Goghari, 2016; Loosli et al., 2016; Yang et al., 2006).

However, cognitive training studies that provide direct evidence of transfer effects are still scarce and the outcomes mixed, albeit the ultimate aim of cognitive training is to go beyond what has been trained on and to eventually improve one's quality of life (Brehmer, et al., 2012; Klingberg, 2010; Richmond, Morrison, Chein, & Olson, 2011). Transfer effects to untrained tasks are typically classified into near and far. In the first case, the untrained task also relies on WM; in the latter case, it relies on other cognitive functions, in addition to WM, such as reasoning, intelligence, attention (Klingberg, 2010).

When targeting transfer effects in relation to WM training, the choice of the transfer task should, in our opinion, be based on two factors: transfer effects after WM training reported in literature studies and the relationship with the trained task. There have been reports on effective WM training improvements in untrained tasks, such as spatial WM, attention, and fluid intelligence in young (Anguera, et al., 2013; Dahlin, Neely, et al., 2008; Dahlin, Nyberg, et al., 2008; Jaeggi et al., 2008, 2010) and older adults (Borella et al., 2010, 2013; Dahlin, Neely, et al., 2008; Dahlin, Nyberg, et al., 2008; Li et al., 2008). Also, significant correlations were found between WM decline in older adults and inhibition and processing speed by Borella, Carretti, and Beni (2008), and between WM and fluid intelligence in terms of the temporary retention of a certain amount of information (Kyllonen & Christal, 1990) and of attentional control processes (Salthouse, Pink, & Tucker‐Drob, 2008). Considering these characteristics, we will verify whether near‐ and far‐transfer effects can be observed following WM training. When using a WM task, we will stay in line with the WM model defined by Baddeley (1992). It refers to a cognitive system that provides temporary storage and manipulation of information necessary to execute complex cognitive tasks. More recently, the WM model proposed by Oberauer (2009) considers WM as “a blackboard for information processing on which we can construct new representations with little interference from old memories” and requires six elements for a WM system, dividing declarative and procedural WM. From our point of view, also supported by Baddeley (2012), this theory is very complex and could be difficult to evaluate experimentally.

Our WM training relies on an *N*‐back task, a WM task introduced by Kirchner (1958) as a visuospatial task with four load factors (“0‐back” to “3‐back”), and by Mackworth (1959) as a visual letter task with up to six load factors. The task involves multiple processes: WM updating, which includes the encoding of incoming stimuli, the monitoring, maintenance, and updating of the sequence, and stimulus matching (matching the current stimulus to the one that occurred N positions back in the sequence). It reflects a number of core executive functions (EFs) besides WM, such as inhibitory control and cognitive flexibility, as well as other higher order EFs such as problem solving, decision making, selective attention (Kane, & Engle, 2002). It has been shown that the *N*‐back task consistently activates dorsolateral prefrontal cortex as well as parietal regions in adult brain (Owen, McMillan, Laird, & Bullmore, 2005). Schneiders, Opitz, Krick, and Mecklinger (2011) have shown that with *N*‐back training it is possible to achieve an improvement in performance and an alteration in brain activity, such as a decreased activation in the right superior middle frontal gyrus (Brodmann area [BA] 6) and posterior parietal regions (BA 40).

The aim of the present study is to verify whether *N*‐back task performance improves during *N*‐back training and EEG recording, and whether transfer effects to other (untrained) cognitive functions can be observed, such as spatial memory, attention, and fluid intelligence, in two different groups of healthy young and older subjects. Although mixed results have been reported (Clark, Lawlor‐Savage, & Goghari, 2017; Lawlor‐Savage & Goghari, 2016; Salminen, Frensch, Strobach, & Schubert, 2015; Stephenson, & Halpern, 2013), in light of the results obtained in previous studies for both near‐ (Li et al., 2008; Stephenson & Halpern, 2013) and far‐transfer effects (Jaeggi et al., 2008, 2010) in healthy young adults and near‐ (Heinzel et al., 2014; Stepankova et al., 2013) and far‐transfer effects (Borella et al., 2010; Heinzel et al., 2016) in healthy older adults, we hypothesize that improvements in the trained task and near‐ and far‐transfer effects are observed in both age‐groups, with greater gains in young compared to older adults. Besides behavioral responses, we will also record ERP responses as they have shown to reflect the time course of cognitive and sensory processes during cognitive task performance. We will thereby focus on the P300, a positive ERP component appearing approximately 300 ms after stimulus presentation, as it has been related to updating WM (Smith‐Spark & Fisk, 2007), to executive functions (Finnigan, O'Connell, Cummins, Broughton, & Robertson, 2011; Zanto, Toy, & Gazzaley, 2010), and to the neural mechanisms behind training‐induced performance changes.

## 2 MATERIALS AND METHODS

### 2.1 Subject recruitment

We recruited 18 healthy young subjects (seven females and 11 males, mean age 26.15 years, range 21–34 years), undergraduate and graduate students and staff of KU Leuven University, and 28 healthy older subjects (15 females and 13 males, mean age 63.11 years, range between 53 and 69 years) were recruited via posters, social media, and the university's Academic Center for General Practice. Participants were healthy, with reported normal or corrected vision, no history of psychiatric or neurological diseases, not on medication, and never participated in WM training (Table 1).

**Table 1:** Demographic data

	Young		Older	
	CTG	PCG	CTG	PCG
Age	25.44 ± 3.32	26.87 ± 3	63.36 ± 4.05	62.86 ± 4.05
Sex	3F (6M)	4F (5M)	8F (6M)	7F (7M)
Education	18 ± 2.87	18.12 ± 3.04	8.64 ± 3.13	8.36 ± 1.55
MMSE	–	–	29.36 ± 0.93	29.5 ± 0.94

CTG: cognitive training group; PCG: passive control group.

### 2.2 Cognitive training program

Healthy younger participants were assigned to two subgroups, cognitive training group (CTG, *N* = 9) and passive control group (PCG, *N* = 9), and healthy older subjects to two subgroups, CTG (*N* = 14) and PCG (*N* = 14), and the results were used to evaluate improvements in WM task performance and to detect transfer effects to other cognitive tasks.

Cognitive training group‐young participants performed WM training (1‐, 2‐, 3‐back task) with visual feedback on the correctness of their behavioral responses and monetary reward (max. 10 €/session), while PCG participants did not undergo any training. We decided to give them not only monetary reward, but also feedback because, according to the self‐determination theory, intrinsic human motivation plays an important role in individuals to be engaged in activities, giving a sense of satisfaction and increasing performance results (Deci & Ryan, 1985). During all training sessions, EEG was recorded.

**Figure 1:**
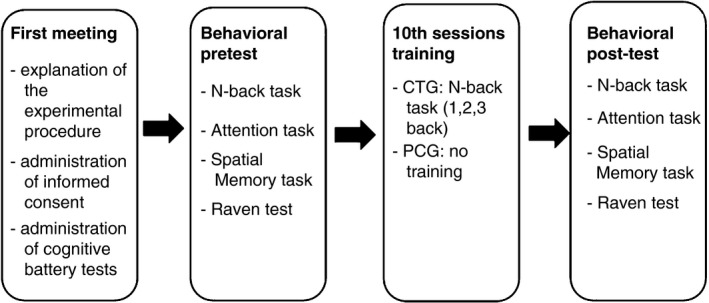
Study design. CTG: cognitive training group; PCG: passive control group

Cognitive training group‐old participants performed the same task that the CTG‐young participants had performed (1‐, 2‐, 3‐back task). Similar to the younger subjects, CTG‐old participants performed WM training with visual feedback on the correctness of their behavioral response and received monetary reward (max 10 €/session). Furthermore, a battery of cognitive tests was administered before and after training (Figure 1) for both groups.

Cognitive training group‐young and older participants performed *N*‐back training during 10 sessions, 3 times per week (during 30 min each), in line with literature reports on significant training and transfer effects after 3 weeks of training (Brehmer et al., 2012; Chein, & Morrison, 2010; Dahlin, Bäckman, Neely, & Nyberg, 2009; Dahlin, Neely, et al., 2008; Dahlin, Nyberg, et al., 2008; Richmond et al., 2011).

In the first experimental session (pretest), each participant was informed about the experimental procedure and invited to sign the informed consent form. The day after the participants performed the behavioral pretest session, and from the third meeting onward, the CTGs (young and older) started the training sessions. The study was approved by our university's ethical committee.

### 2.3 Stimuli

**Figure 2:**
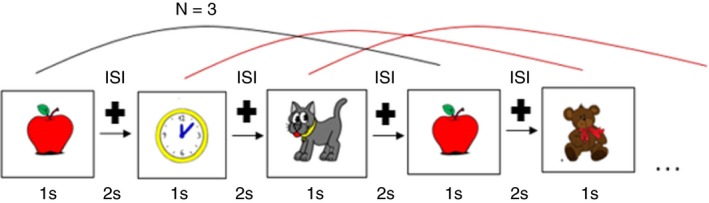
Graphical rendition of 3‐back task

For the *N*‐back stimuli, pictures of meaningful objects were used, presented for 1,000 ms followed by a 2,000‐ms interstimulus interval to which a jitter of ±100 ms was added and during which the picture was replaced by a fixation cross (Figure 2). This was the moment when participants were required to press a button on the keyboard (33% of the pictures were targets). If the response was correct, a green face (visual feedback) appeared on the screen, and if it was wrong, a red face appeared. We opted for colorful pictures that are easy to understand not only for healthy subjects, but also for cognitive decline patients in view of future studies.

The sequences with identical difficulty levels (1‐, 2‐, 3‐back) were grouped into 2‐min. blocks across four sessions. Each session included two repetitions of three sequences with increasing load level (i.e., from 1‐ to 3‐back). In total, there were eight blocks. For each sequence, there were 60 stimuli, presented in pseudorandom order. Before starting with the three sequences, a training session consisting of 10 stimuli for each difficulty level is used to explain our *N*‐back task.

### 2.4 Transfer effect assessment

All participants were administered a battery of pre‐ and post‐tests to evaluate whether there are transfer effects to other cognitive functions (attention, spatial memory, and fluid intelligence) and to assess the effect of using a different version of the *N*‐back task (without visual feedback and nonadaptive). We used Test of Variables of Attention (TOVA; Greenberg, & Waldmant, 1993), visuospatial short‐term WM test (CORSI block‐tapping test; Kessels, Van Zandvoort, Postma, Kappelle, & De Haan, 2000), and RAVEN test for fluid intelligence (Raven & John Hugh Court, 1998). The behavioral pre‐ and post‐tests were administered to compare task performance between groups (CTG and PCG) for the untrained tasks (nonadaptive *N*‐back, TOVA, CORSI, and RAVEN test).

In view of possible transfer effects, it is interesting to note similarities and differences between the *N*‐back task and the CORSI test (Dahlin, Neely, et al., 2008; Dahlin, Nyberg, et al., 2008; Persson, & Reuter‐Lorenz, 2008; Zhao, Wang, Liu, & Zhou, 2011): Both the CORSI and the *N*‐back task measure WM and the capacity for temporarily retaining information, but the CORSI test simply quantifies the spatial span and calls upon the recollection process of previously presented items, while the *N*‐back task is a more complete task as it involves several cognitive processes, including WM updating, and adopts different task rules by using recognition of previously presented items. According to Karbach and Kray (2009), Dahlin, Neely, et al. (2008), Dahlin, Nyberg, et al. (2008), and Li et al. (2008), we should expect the outcomes of near‐transfer task (CORSI and *N*‐back task without visual feedback and nonadaptive) to be different from those of far‐transfer tasks (TOVA and RAVEN test). Cohen's *d* effect sizes (Cohen, Cohen, West, & Aiken, 2003) were reported [*d* = (*M_i_* − *M_j_*)/*SD*_pooled,_ where *M* = mean and *SD*=standard deviation] to indicate the magnitude of the significant differences.

### 2.5 EEG recording

EEG was recorded continuously using a SynAmps RT device (Compumedics, Australia) at a sampling rate of 2 kHz and 32 active Ag/AgCl electrodes: O1, Oz, O2, PO4, PO3, P8, P4, Pz, P3, P7, TP9, CP5, CP1, CP2, CP6, TP10, T7, C3, Cz, C4, T8, FC6, FC2, FC1, FC5, F3, Fz, F4, AF3, AF4, Fp1, and Fp2. The reference electrode was placed at AFz and the ground electrode at CPz.

Before the actual experiment, the subject's electro‐oculogram (EOG) was recorded following the setup of Croft and Barry (2000). The recorded EEG signal was re‐referenced offline to the average of the two mastoid signals (average mastoid reference, TP9 and TP10), corrected for EOG (eye movement and blinking artifacts) using Croft and Barry's (2000) aligned‐artifact average procedure, band‐pass filtered in the 0.1–30 Hz range, and cut into epochs starting from 200 ms pre‐till 1,000‐ms post‐stimulus onset. Baseline correction was performed by subtracting the average of the 200‐ms pre‐stimulus onset activity from the 1,000‐ms post‐stimulus onset activity. Finally, the epochs were downsampled to 100 Hz and stored for ERP detection.

Recorded epochs with incorrect behavioral responses were excluded from further analysis. In addition, epochs with EEG signals >50 µV on any electrode were also excluded. EEG data were analyzed using MATLAB, version R2016a (http://www.nl.mathworks.com/products/matlab/). A three‐way ANOVA (factors *N*‐back × target × session) was used to assess the effect of cognitive training on the P300 amplitude expressed as the difference between target minus nontarget average EEG amplitudes in the 250–500 ms post‐onset time window (area under the curve). Finally, eta‐squared effect sizes (Cohen, 1973) were reported (*η* ^2^ = SS_effect_/SS_total_, where SS = sums of squares) for significant differences.

## 3 RESULTS

### 3.1 Working memory training: Behavioral results

**Figure 3:**
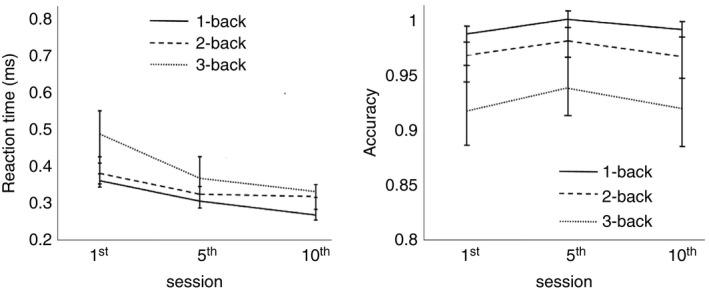
Reaction time and accuracy during cognitive training in CTG (young). RT (left) and accuracy (right) during 10 sessions of cognitive training of younger CTG participants. Error bars indicate *SEM*. CTG: cognitive training group; RT: reaction time

We analyzed the effect of cognitive training by examining behavioral data (accuracy, reaction time [RT]) of our two training groups (CTG) of healthy young and older subjects during 10 sessions of *N*‐back training (Figure 3). Our purpose was twofold: (a) to verify whether training improves *N*‐back task performance, in terms of behavioral responses, and P300 amplitude, after five sessions or whether it is necessary to consider 10 sessions, and (b) to prove that *N*‐back training transfers to other cognitive functions as well.

For CTG‐young participants, we observed RT to decrease with number of training sessions. To test this, we performed a *t* test analysis between the first and middle sessions, between the first and last sessions, and between the middle and last training sessions. We found a significant effect between the first and last sessions for 3‐back (*p* < 0.05, *d* = 1.72) and between the first and middle sessions for 3‐back (*p* < 0.05, *d* = 1.34), confirming that RT decreases significantly between the first and middle sessions compared to the middle and last sessions for which we did not find any significant result. In contrast, when we looked at accuracy, the main effect of session was not significant (*p* = 0.31), indicating that accuracy did not substantially increase as a result of training although there was a trend of improvement between the first and middle sessions. Interestingly, comparing the middle and last sessions, we observed a decrease in performance probably due the boredom of the young subjects.

**Figure 4:**
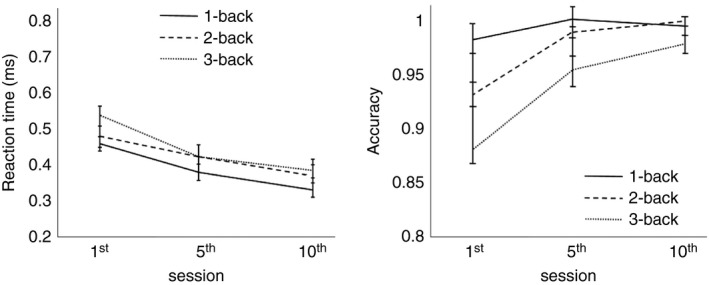
RT and accuracy during cognitive training in CTG (old). RT (left) and accuracy (right) during 10 sessions of cognitive training of older CTG participants. Error bars indicate *SEM.* CTG: cognitive training group; RT: reaction time

We also examined accuracy and RT during *N*‐back training of older adults of CTG (Figure 4). We performed a *t* test analysis between the first and middle sessions, the first and last session, and the middle and last sessions. We found for RT a significant effect between first and last sessions for 1‐back (*p* < 0.05, *d* = 1.06), for 2‐back (*p* < 0.05, *d* = 0.84), and for 3‐back (*p* < 0.01, *d* = 0.96), and between first and middle sessions for 3‐back task (*p* < 0.05, *d* = 1.12), indicating that the subjects become faster with training, especially in the first five sessions. We did not find any significant differences between the middle and last training sessions. For the accuracy, we found significant effects between the first and last sessions of 2‐back (*p* = 0, *d* = 0.59) and 3‐back (*p* = 0, *d* = 0.92), and between the first and middle sessions of 2‐back (*p* < 0.001, *d* = 0.51) and 3‐back (*p* < 0.01, *d* = 0.64). We did not find any significant difference between the middle and last sessions, indicating that accuracy improves with training, mostly for the 2‐ and 3‐back tasks and between the first and middle sessions.

### 3.2 Working memory training: EEG results

**Figure 5:**
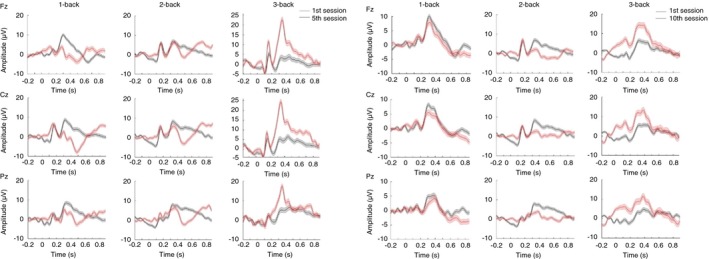
Event‐related potentials (ERPs) during the training for cognitive training group (CTG young). P300 amplitude difference (target minus nontarget) shown for channels Fz, Cz, and Pz for the first (black curves) and middle sessions (red curves) (left three columns) and first (black curves) and last (red curves) sessions (right three columns) of nine young adults of the CTG. Significance was measured using three‐way ANOVA (*p* < 0.01). Error bars indicate *SEM*

**Figure 6:**
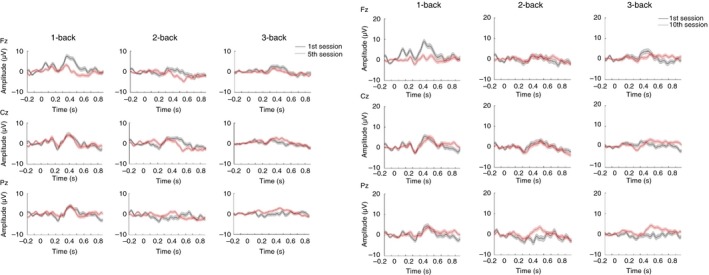
Event‐related potentials (ERPs) during the training for cognitive training group (CTG; older). P300 amplitude difference (target minus nontarget) shown for channels Fz, Cz, and Pz and for the first (black curves) and middle sessions (red curves) (left three columns) and first (black curves) and last (red curves) sessions (right three columns) of 14 older adults of the CTG. Significance was measured using three‐way ANOVA (*p* < 0.01). Error bars indicate *SEM*

As neuroimaging studies have shown that, during *N*‐back task performance, the most activated brain regions are the lateral premotor cortex, dorsal cingulate and medial premotor cortex, dorsolateral and ventrolateral prefrontal cortex, frontal poles, and medial and lateral posterior parietal cortex (Gevins et al., 1990), and that the midline electrodes are the most significant ones (Mahncke et al., 2006; Watter, Geffen, & Geffen, 2001), we decided to analyze ERPs, more specifically the P300, using electrodes located over these areas: Fz, Pz, and Cz. Figures 5 and 6 show P300 amplitudes (250–400 ms) in three different sessions during training (first, middle, and last sessions) for CTG in young and older adults.

P300 amplitudes of midline electrodes (Fz, Cz, Pz) were compared using a three‐way ANOVA (*N*‐back × target × session). Significant differences between pre‐ and post‐training sessions were found in healthy young subjects for the interaction between first and middle sessions × target for 3‐back in channel Fz, *F*(1) = 7.2620, *p* < 0.01, *η* ^2^ = 2.68%, in channel Cz, *F*(2) = 6.0811, *p* < 0.05, *η* ^2^ = 0.81%, and in channel Pz, *F*(1) = 5.4272, *p* < 0.05, *η* ^2^ = 22.82%, and for the interaction between first and last sessions × target for 3‐back in channel Fz, *F*(1) = 6.4155, *p* < 0.05, *η* ^2^ = 17.46%, and in channel Cz, *F*(1) = 3.9479, *p* < 0.05, *η* ^2^ = 6.25%. Furthermore, P300 amplitude was higher for the *N*‐back levels that were easier (1 and 2‐back) and lower for the more difficult one (3‐back), especially when comparing the first and last training sessions. The P300 amplitude was largest for the frontal electrode (Fz) and decreased for the central (Cz) and posterior electrodes (Pz) for the 1‐back task (Figure 5). The P300 amplitude decreased progressively from the easiest task (1‐back) to the most difficult one (3‐back), although for the most difficult task, as a result of WM training, it increased in the last session of training compared to the 1‐ and 2‐back tasks. Taken together, these data support the observation that the P300 amplitude decreases with increased task load/difficulty, but that with *N*‐back training, it is possible to inverse the process as revealed by an increased P300 amplitude for the 3‐back task compared to the easier ones (1‐ and 2‐back).

We also analyzed P300 amplitudes of the midline electrodes (Fz, Cz, Pz) with a three‐way ANOVA (*N*‐back, target, and session) for CTG‐old participants. We found significant effects for the interaction between first and middle sessions × target for 3‐back in channel Pz, *F*(1) = 4.2120, *p* < 0.05, *η* ^2^ = 8.15%, and for the interaction between the first and last sessions x target for 3‐back in channel Pz, *F*(1) = 14.2780, *p* < 0.001, *η* ^2^ = 11.84%. Compared to the healthy young subjects, the P300 amplitude (target minus nontarget) was significant in the parietal area, while for young subjects, it was in frontal and central areas. Furthermore, the P300 amplitude was higher for the *N*‐back tasks that were easier (1‐ and 2‐back) and lower for the more difficult one (3‐back). In this case, after training the older adults, the P300 amplitude increases for the most difficult task (3‐back), showing that the P300 amplitude decreases with increasing task load/difficulty and that *N*‐back training can change the neural response of the subject (Figure 6). These findings confirm the results of Gevins and Smith (2003) who reported that training on an *N*‐back task shows EEG changes in responses to changes in the mental effort required for task performance.

Salminen et al. (2016) showed for both young and older subjects benefits after a *N*‐back training based on behavioral responses, with different degrees of improvement for older adults. Friedman and Simpson (1994) found differences in ERP amplitudes of young and older adults during oddball task performance. Given these observations, we looked for differences in the P300 components of young and older subjects. The results showed significant differences between age‐groups x target in the first training session for the 2‐back task in channel Fz, *F*(1) = 13.8222, *p* < 0.01, *η* ^2^ = 5.68%, in channel Cz, *F*(1) = 6.3675, *p* < 0.05, *η* ^2^ = 1.05%, and in channel Pz, *F*(1) = 10.1196, *p* < 0.001, *η* ^2^ = 20.26%, and in the last training session for the 3‐back task in channel Fz, *F*(1) = 20.1882, *p* < 0.001, *η* ^2^ = 9.56%, in channel Cz, *F*(1) = 17.5405, *p* < 0.001, *η* ^2^ = 9.75%, and in channel Pz, *F*(1) = 11.6941, *p* < 0.001, *η* ^2^ = 12.45%. We did not find any significant difference between young and older adults in the middle training session.

### 3.3 Transfer effects (pre‐ and post‐tests)

**Figure 7:**
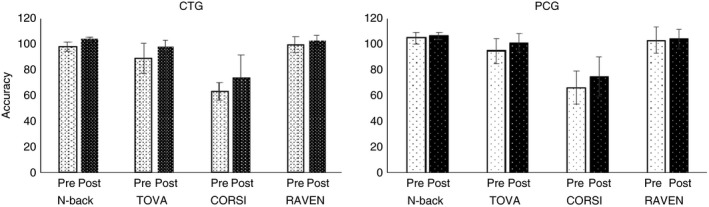
Accuracy pre–post tests (TOVA, RAVEN, CORSI) for two groups of young adults (CTG and PCG). Pre‐ and post‐test performance (in % correct responses) of young adults of the CTG (left) and PCG (right) for the *N*‐back task, TOVA, CORSI, and RAVEN. Error bars indicate *SEM*. CTG: cognitive training group; PCG: passive control group

**Figure 8:**
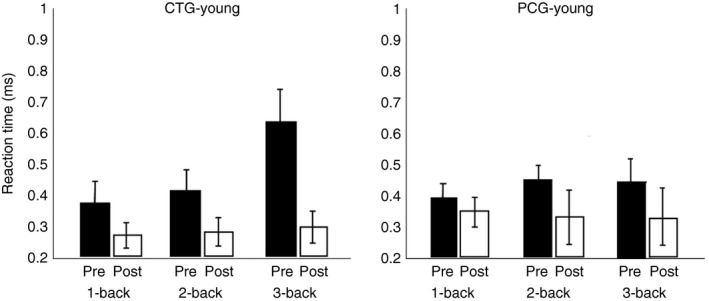
Reaction time (RT) pre–post test in *N*‐back for two groups of young adults (CTG and PCG). Pre‐ and post‐test RT (correct responses only) for the *N*‐back task of young adults of the CTG (left) and PCG (right). Error bars indicate *SEM*. CTG: cognitive training group; PCG: passive control group

Percent correct responses (means and standard deviation) for each task are presented in Tables 2 and 3 for healthy young and older subjects, for pre‐ and post‐tests. We did not find any significant intragroup differences in pretest performance for the healthy young and older adults, while we found significant intergroup differences between healthy young and older adults. A *t* test analysis showed significant differences between healthy young and older adults in both CTGs for *N*‐back task (*p* < 0.001, *d* = 5.56) and RAVEN (*p* < 0.01, *d* = 1.95) and PCGs for *N*‐back task (*p* < 0.05, *d* = 2.21) and RAVEN (*p* < 0.05, *d* = 1.77). A *t* test analysis was conducted between groups (CTG and PCG) and between sessions (pretest and post‐test, Figures 7 and 8) for healthy young and older subjects for the *N*‐back, TOVA, CORSI, and RAVEN tests. For young subjects (Figure 7), significant differences were observed between pre–post tests for accuracy for CTG in *N*‐back task (*p* < 0.001, *d* = 2.13), TOVA (*p* < 0.05, *d* = 0.95), CORSI (*p* < 0.05, *d* = 1.14) and RAVEN (*p* < 0.05, *d* = 0.52), and for PCG in TOVA (*p* < 0.05, *d* = 0.43) and CORSI (*p* < 0.05, *d* = 0.62). Furthermore, comparing the two groups (CTG and PCG), we found significant differences for *N*‐back task (*p* < 0.05, *d* = 1.89). No significant differences in TOVA, CORSI, and RAVEN test accuracies between groups were found. Comparing pre–post *N*‐back task results (Figure 8), significant effects were found for RT between CTG and PCG (*p* < 0.05, *d* = 0.89).

**Table 2:** Pre‐ and post‐test performance (accuracy) in percent correct responses (means and standard deviation) of training (*N* = 9) and passive control groups (*N* = 8) for trained (*N*‐back) and untrained tasks of young healthy subjects

Task	Cognitive training group		Passive control group	
	Pretest	Post‐test	Pretest	Post‐test
*N*‐back task	93.07 ± 3.56	98.63 ± 1.36	93.71 ± 4.05	95.12 ± 2.30
TOVA task	84.22 ± 11.33	93.11 ± 4.48	84.75 ± 8.55	90.25 ± 6.36
CORSI task	59.99 ± 6.66	70.32 ± 16.78	59.16 ± 11.51	66.66 ± 13.80
RAVEN test	94.63 ± 5.76	97.40 ± 4.01	92 ± 8.98	92.70 ± 7.01

**Table 3:** Pre‐ and post‐test performance (accuracy) of training (*N* = 14) and passive control groups (*N* = 14) of older healthy subjects. Conventions are as in Table 2

Task	Cognitive training group		Passive control group	
	Pretest	Post‐test	Pretest	Post‐test
*N*‐back task	15.05 ± 6.96	69.81 ± 7.34	22.48 ± 6.49	41.03 ± 8.92
TOVA task	95.33 ± 0.67	97 ± 0.61	93.68 ± 1.42	79.58 ± 6.69
CORSI task	72 ± 6.13	73 ± 3.48	77.5 ± 3.75	85.83 ± 3.81
RAVEN test	67.33 ± 6.54	80 ± 2.57	79.72 ± 3.70	87.22 ± 2.01

CTG: cognitive training group; PCG: passive control group.

**Figure 9:**
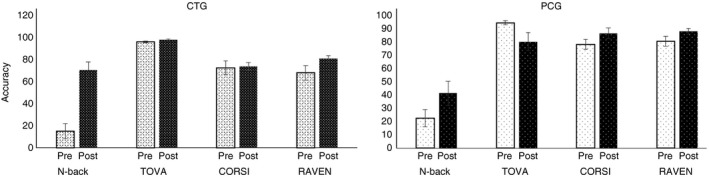
Accuracy pre–post tests (TOVA, RAVEN, CORSI) for two groups of older adults (CTG and PCG). Pre‐ and post‐test performance (in % correct responses) of older adults of the CTG (left) and PCG (right) for the *N*‐back task, TOVA, CORSI, and RAVEN. Error bars indicate *SEM*. CTG: cognitive training group; PCG: passive control group

**Figure 10:**
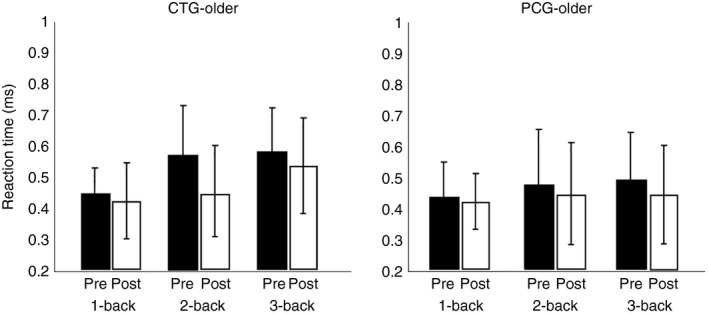
Reaction time (RT) pre–post test in *N*‐back for two groups of older adults (CTG and PCG). Pre‐ and post‐test RT (correct responses only) for the *N*‐back task of older adults of the CTG (left) and PCG (right). Error bars indicate *SEM.* CTG: cognitive training group; PCG: passive control group

Furthermore, a *t* test was used for healthy older subjects comparing pretest and post‐tests performances in CTG, PCG, and between groups (CTG and PCG) (Figures 9 and 10). Our results showed significant differences for accuracy for CTG in *N*‐back task (*p* < 0.001, *d* = 2.3), TOVA (*p* < 0.001, *d* = 0.78), and RAVEN (*p* < 0.05, *d* = 0.74). No significant differences were found for PCG. The comparison between CTG and PCG showed significant results for *N*‐back task (*p* < 0.01, *d* = 0.59), TOVA (*p* < 0.05, *d* = 0.17), and CORSI (*p* < 0.01, *d* = 0.06). No significant differences in RAVEN for accuracy were found between groups (Figure 9). Significant effects were found for RT between CTG and PCG for pre–post *N*‐back task (*p* < 0.05, *d* = 0.48) (Figure 10).

Since Dahlin, Neely, et al. (2008), Dahlin, Nyberg, et al. (2008) showed that younger adults benefit more from cognitive training than older adults and Bherer et al. (2005) showed the opposite (older subjects gained more positive effects than younger ones), we also analyzed the differences between young and older adults for CTG and for PCG separately. We used a *t* test for the factors age‐group (young and older) and pre–post training. Significant pre–post training differences were found in accuracy for CTG (young vs. older) for TOVA (*p* < 0.05, *d* = 1.08) and CORSI (*p* < 0.05, *d* = 1.1), and for PCG (young vs. older) for TOVA (*p* < 0.05, *d* = 1.57) and CORSI (*p* < 0.05, *d* = 2.1). Finally, we considered Pearson's correlation between P300, accuracy, and RT. Our results revealed significant correlations in healthy older adults for the interaction of 1‐back P300 and RT (*p* < 0.05, *r* = 0.49783), for the interaction of 2‐back P300 and RT (*p* < 0.0001, *r* = 0.96356), and for the interaction of 3‐back P300 and RT (*p* < 0.05, *r* = 0.52979) in channel Fz; for the interaction of 2‐back P300 and RT (*p* < 0.001, *r* = 0.89268) and for the interaction of 3‐back P300 and RT (*p* < 0.001, *r* = 0.70583) in channel Cz; and finally for the interaction of 1‐back P300 and RT (*p* < 0.01, *r* = 0.55589) and for the interaction of P300 and RT (*p* = <0.001, *r* = 0.40885) in channel Pz. Also, we found significant correlations for the interaction between accuracy and 1‐back P300 (*p* < 0.01, *r* = 0.57601), 2‐back P300 (*p* < 0.001, *r* = 0.96825), and 3‐back P300 in channel Fz (*p* < 0.01, *r* = 0.59276); for the interaction between accuracy and 1‐back P300 (*p* < 0.05, *r* = 0.950942), 2‐back P300 (*p* < 0.001, *r* = 0.90043), and 3‐back P300 (*p* < 0.001, *r* = 0.76722) in channel Cz; and for the interaction between accuracy and 1‐back P300 (*p* < 0.01, *r* = 0.65725), 2‐back P300 (*p* < 0.001, *r* = 0.86757), and 3‐back P300 (*p* < 0.05, *r* = 0.47328) in channel Pz. While significant correlations were found between P300 and both accuracy and RT for older adults, we found a significant correlation only between RT and P300 for young adults. Our results showed significant interactions between RT and 1‐back P300 (*p* < 0.001, *r* = −0.99202) and between RT and 3‐back (*p* < 0.01, *r* = 0.89219) in channel Cz; and between RT and 1‐back (*p* < 0.05, *r* = −0.764) and between RT and 3‐back P300 (*p* < 0.001, *r* = 0.9716) in channel Pz.

## 4 DISCUSSION

The main purpose of our study was to investigate whether cognitive training improves only trained task performance or also transfers to other cognitive tasks. To verify this, we performed a study where we subjected a group of healthy young and older subjects to 10 *N*‐back training sessions, and assessed their performance on a battery of untrained cognitive tasks (different version of *N*‐back, TOVA, CORSI, and RAVEN tests) before and after training. To assess whether level of task difficulty affected training outcome, we considered groups of young and older participants (CTG) that performed the 1‐, 2‐, 3‐back version of the *N*‐back task and other groups of young and older participants (PCG) that performed no training but were subjected to the same pre‐ and post‐test battery. We found for our CTG of healthy young subjects that training indeed improves *N*‐back task performance compared to PCG. Additionally, their transfer effects to other untrained tasks were significant for near‐transfer tasks using an untrained, nonadaptive version of the *N*‐back task (WM task) and for a far‐transfer task, RAVEN, that measures fluid intelligence, also compared to PCG. Furthermore, also Cohen's *d* confirmed the large effects after WM training in the pre‐ and post‐tests. As mentioned above, transfer effects were found for tasks that overlap in terms of cognitive processes involved, for the untrained *N*‐back version, in WM ability (Dahlin, Nyberg, et al., 2008), and for the RAVEN test, in temporary information retention and attentional control processes (Jaeggi et al., 2008). These results showed clearly that it was not a test–retest effect as PCG did not show any significant differences in pre‐ and post‐tests. Furthermore, it was surprising to see that the CORSI test, in our case a measure of near‐transfer for WM, did not indicate any significant improvement after training. One possible explanation is that the *N*‐back training performance did not involve a spatial component. For our participants, it turned out to be more difficult to allocate attention to spatial differences, as the CORSI test requires. In contrast, the untrained *N*‐back transfer task differed in feedback and task difficulty level, but the stimuli were only visual, not spatial, as for the trained task. Additionally, although the TOVA shares the attentional process with the trained task, it also involves a spatial process that was not trained for. For our healthy older subjects, we found significant improvements in *N*‐back, TOVA, and RAVEN test performance for CTG compared to PCG. Also, these results support the aforementioned theory of cognitive processes overlapping different tasks. In addition, although unexpected, we found a significant difference in TOVA for attention. The significance of these findings was clearer for older subjects compared to young adults because attentional control decreases with age (Karbach & Verhaeghen, 2014), and there is more room for improvement compared to young individuals. We do not think that experimental conditions, in terms of task or training features and the presence of the researcher, might have affected these results, as we kept the same conditions in each session for both groups. We do believe that individual characteristics, such as expectation and motivation, could have affected our results (cf. Deci & Ryan, 1985). These results are in line with the studies of Yang et al. (2006) and Bherer et al. (2005) who showed that, also in the aging brain, the capacity of plasticity improves cognitive functioning. However, the present findings are in contrast with other studies that showed greater improvements for young subjects compared to older subjects (Dahlin, Neely, et al., 2008). In general, we found that both young and older adults achieved gains in *N*‐back task performance after training, but this did not transfer to the same extent to untrained functions, as for older adults we reported improvements in *N*‐back (near‐transfer effect) and RAVEN (far‐transfer effect) and also in TOVA (far‐transfer effect), while in young adults only in *N*‐back and RAVEN, indicating greater improvements for older adults (Bherer et al., 2005). We verified our initial hypothesis by showing improvements in the trained task and near‐ and far‐transfer effects, although it was surprising to find a larger gain for older adults compared to young subjects (Dahlin, Neely, et al., 2008; Dahlin, Nyberg, et al., 2008), and no transfer to CORSI although it is a near‐transfer task (Dahlin, Nyberg, et al., 2008; Klingberg, 2010).

Additionally, we tested whether differences in P300 amplitude were visible after five sessions or whether it was necessary to consider 10 sessions. We observed significant differences in P300 amplitude between first and middle sessions and between first and last sessions (target minus nontarget) for young and older subjects, complementing the study of Friedman and Simpson (1994) who used a simple oddball paradigm to observe differences in ERP amplitude between young and older adults. Our results showed a higher P300 for young adults in frontal, central, and parietal areas, especially for the first five sessions. The P300 of the most difficult *N*‐back task (3‐back) was strongest affected by training as it increased in the last session of training compared to the easier tasks, 1‐ and 2‐back. Also, older adults showed a higher P300 amplitude after five and ten sessions of *N*‐back training, and no significant differences between the middle and last sessions. Compared to the healthy young subjects, the P300 amplitude was stronger over the parietal area. As for young adults, P300 amplitude became higher for the most difficult task, showing the effectiveness of *N*‐back training. These findings for both training groups showed significant differences between the first five sessions, but not for the last five sessions, suggesting that five training sessions could be enough to reach significant improvements in P300 for the trained task in healthy adults. In general, our initial hypothesis was verified as we measured an increase in P300 amplitude with training, indicating that the task was easier for the participants. In particular, the most significant effect was found for the highest difficult level, the 3‐back task, suggesting a large improvement in storage, manipulation, and updating processes involved in the *N*‐back task.

In summary, in light of our results, an issue that deserves further consideration is why *N*‐back training in the study with young and older adults did not produce significant near‐transfer effects in a similar task (CORSI), whereas in contrast, Dahlin, Neely, et al. (2008), Dahlin, Nyberg, et al. (2008) observed a near‐transfer effect to another memory task. We hypothesize that this might be due to specific task‐trained features and strategies developed by the participants during training that could help in storage and manipulation information.

## 5 CONCLUSION

Studying cognitive plasticity across different epochs in one's life span has become very important given the steadily increasing life expectancy. We decided to investigate the potential of cognitive training to compensate for age‐related cognitive decline and provided evidence of beneficial effects, both in healthy young and in older subjects. In addition, the cognitive decline in WM is also supported by decline in the frontoparietal regions that have an important role in WM (Rajah & D'Esposito, 2005). We noticed differences in P300 responses for young and older adults and showed that *N*‐back training not only improves WM but also transfers to attention and fluid intelligence for young and older adults. These results provide evidence for brain plasticity, in particular in older adults, although the degree and extent of it are expected to decrease with age. In the future, we want to repeat the same experiment with older adults performing a multisensory *N*‐back task, more specifically a dual (visual and auditory) *N*‐back task, as Salminen et al. (2016) found larger improvements compared to single *N*‐back task in young subjects, and their use of specific strategies during task performance. It remains to be seen whether these tasks can be used in practice to maintain or even improve quality of life across ages.
